# A Bird’s Eye View of the Systematics of Convolvulaceae: Novel Insights From Nuclear Genomic Data

**DOI:** 10.3389/fpls.2022.889988

**Published:** 2022-07-14

**Authors:** Ana Rita G. Simões, Lauren A. Eserman, Alexandre R. Zuntini, Lars W. Chatrou, Timothy M. A. Utteridge, Olivier Maurin, Saba Rokni, Shyamali Roy, Félix Forest, William J. Baker, Saša Stefanović

**Affiliations:** ^1^Royal Botanic Gardens, Kew, Richmond, United Kingdom; ^2^Conservation & Research Department, Atlanta Botanical Garden, Atlanta, GA, United States; ^3^Systematic and Evolutionary Botany Lab, University of Ghent, Ghent, Belgium; ^4^Department of Biology, University of Toronto Mississauga, Mississauga, ON, Canada

**Keywords:** *Ipomoea*, *Convolvulus*, *Cuscuta*, phylogeny, classification, Angiosperms353

## Abstract

Convolvulaceae is a family of c. 2,000 species, distributed across 60 currently recognized genera. It includes species of high economic importance, such as the crop sweet potato (*Ipomoea batatas* L.), the ornamental morning glories (*Ipomoea* L.), bindweeds (*Convolvulus* L.), and dodders, the parasitic vines (*Cuscuta* L.). Earlier phylogenetic studies, based predominantly on chloroplast markers or a single nuclear region, have provided a framework for systematic studies of the family, but uncertainty remains at the level of the relationships among subfamilies, tribes, and genera, hindering evolutionary inferences and taxonomic advances. One of the enduring enigmas has been the relationship of *Cuscuta* to the rest of Convolvulaceae. Other examples of unresolved issues include the monophyly and relationships within Merremieae, the “bifid-style” clade (Dicranostyloideae), as well as the relative positions of *Erycibe* Roxb. and Cardiochlamyeae. In this study, we explore a large dataset of nuclear genes generated using Angiosperms353 kit, as a contribution to resolving some of these remaining phylogenetic uncertainties within Convolvulaceae. For the first time, a strongly supported backbone of the family is provided. *Cuscuta* is confirmed to belong within family Convolvulaceae. “Merremieae,” in their former tribal circumscription, are recovered as non-monophyletic, with the unexpected placement of *Distimake* Raf. as sister to the clade that contains Ipomoeeae and *Decalobanthus* Ooststr., and Convolvuleae nested within the remaining “Merremieae.” The monophyly of Dicranostyloideae, including *Jacquemontia* Choisy, is strongly supported, albeit novel relationships between genera are hypothesized, challenging the current tribal delimitation. The exact placements of *Erycibe* and *Cuscuta* remain uncertain, requiring further investigation. Our study explores the benefits and limitations of increasing sequence data in resolving higher-level relationships within Convolvulaceae, and highlights the need for expanded taxonomic sampling, to facilitate a much-needed revised classification of the family.

## Introduction

Convolvulaceae are a cosmopolitan plant family, widespread across tropical and temperate regions. It includes important food crops such as sweet potato (*Ipomoea batatas* L.) and water spinach (*Ipomoea aquatica* Forssk.) as well as a range of ornamental plants, including morning glories [e.g., *Ipomoea tricolor* Cav., *Ipomoea indica* (Burm.) Merr., *Ipomoea nil* (L.) Roth] and bindweeds (*Convolvulus* L. and *Calystegia* R. Br.). This family also harbors genus *Cuscuta* L. (dodders), members of which are stem parasites with little to no chlorophyll, representing one of 12 independent origins of haustorial parasitism across angiosperms (Nickrent, 2020). Convolvulaceae are phytochemically very diverse, containing a range of alkaloids, reflected in its wide array of phytotherapeutical and medicinal applications (Eich, 2008). The family is also well known to produce ergot alkaloids in the seeds of some species of *Argyreia* Lour. and *Ipomoea* L., as a result of an association with the fungus *Periglandula*, analogous to the infection of grasses by other related fungi (Beaulieu et al., 2021).

Convolvulaceae include 1,977 accepted species, classified into 60 genera and 12 tribes (Stefanović et al., 2003; Staples and Brummitt, 2007; POWO, 2022). The distribution of the species across genera and tribes is markedly uneven, with almost half of the diversity of the family being concentrated in tribe Ipomoeeae alone (835 species), followed by Convolvuleae (242 species), and Cuscuteae (218 species). These three tribes have been widely sampled in recent molecular phylogenetic studies (García et al., 2014; Williams et al., 2014; Muñoz-Rodríguez et al., 2019) and all three were shown to be monophyletic. However, the remaining nine tribes, containing 682 species distributed across 46 genera, are far less studied.

Classifications from before the onset of molecular phylogenetics and the application of monophyly as the primary principle of classification (Backlund and Bremer, 1998) lack the predictive power that phylogenetic classifications have, due to the absence of a connection between systematic arrangements and their shared, derived characters. In Convolvulaceae, as in many other plant families, the introduction of molecular phylogenetic approaches has brought novel perspectives on the classification systems, and has allowed the re-circumscription of higher-level taxonomic ranks (e.g., subfamilies, tribes, etc.). This improves the predictive power of the classification, and accelerates taxonomic progress.

The first family level phylogeny was produced based on plastid markers (*rbcL*, *atpB*, *psbE-J* operon, and *trnL-trnF* intron/spacer), sampling 52 of the 60 genera recognized at the time (Stefanović et al., 2002). Although the number of genera is currently the same, since this study there have been systematic changes, whereby some genera were synonymized with others, and new ones described (e.g., Staples, 2006; Buril et al., 2013, 2015; Simões and Staples, 2017; Petrongari et al., 2018; Simões and More, 2018; Simões et al., 2020; Staples et al., 2020) (Supplementary Table 1).

Stefanović et al. (2002) were the first to demonstrate the monophyly of Convolvulaceae, as well as of its three subfamilies as recognized at the time – Convolvuloideae, Humbertioideae, and Cuscutoideae – while exposing the non-monophyly of several tribes and genera across the family. Five of the tribes recognized at that time were clearly shown as non-monophyletic (Convolvuleae, Merremieae, Cresseae, Poraneae, Erycibeae), leading to a revision of the tribal classification that would reflect the newly recovered relationships (Stefanović et al., 2003). No subfamilies were formally proposed, but six large clades were identified which can be converted into informal subfamilies: Convolvuloideae, Dicranostyloideae (also called the “bifid style clade”), Cuscutoideae, Eryciboideae, Cardiochlamyoideae, and Humbertioideae. As for tribal ranking, 12 tribes were recognized, among which tribe “Merremieae” was the only one not confirmed to be monophyletic and left as of uncertain placement within the family (Stefanović et al., 2003).

A substantially expanded sampling of this group allowed for re-circumscription of its largest genus, *Merremia* Dennst. ex Endl. (Simões et al., 2015). It was found to be polyphyletic, and subsequently was divided into ten monophyletic genera, all of which are morphologically diagnosable and have received moderate-to-strong support in molecular phylogenies. However, while this study made significant progress in the generic circumscription within the tribe, the data and phylogenetic analyses were not robust enough to test the monophyly of the group itself, nor to demonstrate the relationships between the genera. Three of the genera were suggested to be sister to tribe Ipomoeeae (*Merremia* s.s., *Daustinia* Buril & A. R. Simões, and *Decalobanthus* Ooststr.), while the exact placement of the other seven have remained unresolved.

At the higher classification level, the “bifid style” clade, Dicranostyloideae, is the least resolved, with most of the relationships between its genera remaining uncertain. Also, their generic circumscription is questionable, with several of the genera (e.g., *Bonamia* Thouars, *Calycobolus* Willd. ex Schult.) having already been demonstrated not to be monophyletic (Stefanović et al., 2002). The same is true for *Jacquemontia*, a genus with a shortly divided style and ellipsoid stigmas, previously placed in tribe Convolvuleae (in the “single-style” clade, Convolvuloideae). As one of the most surprising results based on molecular phylogenetic evidence, this lineage has been moved to Dicranostyloideae, and assigned to its own tribe, Jacquemontieae (Stefanović et al., 2003). Interestingly, *Jacquemontia* seemed to be sister to the rest of Dicranostyloideae, but this relationship was only weakly supported (Stefanović and Olmstead, 2004) and has remained to be confirmed.

Finally, as one of the largest outstanding enigmas in Convolvulaceae, the phylogenetic position of *Cuscuta* within the family remains uncertain, owing largely to the rapid molecular evolution observed in this parasitic genus, across all three genomes, and accompanying analytical difficulties this entails (Stefanović and Olmstead, 2004). Based on the sequence data derived from all three plant genomes, at least two non-parasitic lineages are shown to diverge within the Convolvulaceae before *Cuscuta*. However, the exact sister group of *Cuscuta* could not be ascertained, even though many alternatives were rejected with confidence (Stefanović and Olmstead, 2004).

Recently, significant progress has been made in the incorporation of genomic data studies in tribes Ipomoeeae (Eserman et al., 2014; Wu et al., 2018; Muñoz-Rodríguez et al., 2019) and Cuscuteae (Banerjee and Stefanović, 2019, 2020), with a clear benefit in providing a stronger framework for comparative work, e.g., estimating divergence times (Eserman et al., 2014; Carruthers et al., 2020), chromosome evolution (Ibiapino et al., 2022), etc. Higher-level organellomic approach, with an initial sampling across the family, uncovered some unusual scenarios of organellar evolution, especially regarding their mitogenomes, but the monophyly of “Merremieae” and the sister group of *Cuscuta* remained uncertain (Lin et al., 2022). However, most of the family has not yet caught up with these novel methodologies, which could finally bring clarity to the backbone relationships within Convolvulaceae, and unlock progress in systematics, biogeographic, and evolutionary studies at the level of the entire family.

A novel approach that has revolutionized evolutionary and systematic studies in the phylogenomic era is target sequence capture, in which genomic libraries are enriched for a specific set of genes (Dodsworth et al., 2019). In plants, the development of the Angiosperms353 (Johnson et al., 2019), a universal target capture probe set, has allowed standardization of genomic data generated for phylogenetic inference in angiosperms (Baker et al., 2021), enabling easier combination of different datasets and materials, including old herbarium specimens that are proven to be great source of genetic data (Brewer et al., 2019). This probe set has demonstrated its potential to advance phylogenetic studies and significantly resolve relationships with outstanding uncertainty across different taxonomic levels, from ordinal (e.g., Commelinales: Zuntini et al., 2021; Cornales: Thomas et al., 2021; Dipsacales: Lee et al., 2021) to familial (Orchidaceae: Eserman et al., 2021; Pérez-Escobar et al., 2021; Cyperaceae: Larridon et al., 2021) and even infra-generic levels (Shee et al., 2020; Slimp et al., 2021). Based on this universal probe set, large collaborative efforts such as Plant and Fungal Tree of Life Project (PAFTOL, Baker et al., 2022^1^) and Genomics for Australian Plants (GAP^2^) have generated an incomparable amount of genomic data for nearly all families and more than seven thousand genera of flowering plants, on which relationships among all plant families of angiosperms are being analyzed, at taxonomic and phylogenetic scales never attempted before. This unique and comprehensive dataset has an additional feature. The genes targeted by the Angiosperms353 probes are nuclear, which, in contrast to previous studies relying on plastid markers (Stefanović et al., 2002), is expected to facilitate the inclusion of parasitic species in broader analyses, given the documented gene loss tendency in plastid genomes, as observed in *Cuscuta* (Braukmann et al., 2013; Banerjee and Stefanović, 2019, 2020).

In the present study, we make use of these recently available nuclear genomic data towards: (1) exploring the deeper relationships between the main clades within Convolvulaceae; (2) testing the monophyly of tribes, insofar as the taxon sampling allows it; and (3) resolving the position of *Cuscuta* in relation to the remaining of Convolvulaceae.

## Materials and Methods

### Taxon Sampling and Outgroup Selection

Genomic data of 34 out of the 60 genera of Convolvulaceae were analyzed, covering initially all 12 tribes (sensu Stefanović et al., 2003). A targeted effort was made to represent the main lineages in the family as informed by the molecular phylogenies of Stefanović et al. (2002) and Simões et al. (2015), as well as to include the most morphologically divergent genera. In our current study, half of the tribes are represented by a single species (Cuscuteae, Jacquemontieae, Aniseieae, Maripeae, Erycibeae, and Humbertieae). Therefore, the monophyly of these tribes cannot be assessed by our current data, but their relationships to each other and with other tribes within Convolvulaceae can be evaluated, commensurate with our “top-down” phylogenetic approach and focus of this paper. Other six tribes are represented by 2–8 species or genera allowing to assess not only their relationships with the remainder of the family but also their monophyly. Our sampling was limited to one species per genus except for the exceptionally large genus *Ipomoea*, which was represented with two species. Two other species within the family were excluded from further analysis: *Keraunea capixaba* Lombardi, for which preliminary analyses suggested a placement outside of Convolvulaceae; and *Anisea martinicensis* (Jacq.) Choisy, for insufficient data in comparison to the remaining samples. The only data available for *Convolvulus* is transcriptome data from the OneKP Project (One Thousand Plant Transcriptomes Initiative, 2019). Therefore, this genus was included in the analysis of exon data but excluded from supercontig analyses. Complete list of taxa and samples is provided in Supplementary Table 2.

Previous phylogenetic analyses have demonstrated that Convolvulaceae, including the parasitic genus *Cuscuta*, are monophyletic, and that *Humbertia madagascariensis* Lam. is sister to the rest of the family (Stefanović et al., 2002; Stefanović and Olmstead, 2004). We tested these hypotheses using a dataset of 35 species sampled from Convolvulaceae, five from the sister family Solanaceae, and one species from Montiniaceae, as the most distant outgroup in Solanales. Because alignments included individuals from three families, only exon sequences were used to estimate this phylogeny to improve homology assessment in alignment (Supplementary Figure 1).

### DNA Extraction and Target Sequence Capture

Data production followed the protocol outlined in Baker et al. (2022). DNA samples were obtained from herbarium collections at Royal Botanic Gardens, Kew (K) or Kew’s DNA Bank. DNA was isolated using the CTAB method (Doyle and Doyle, 1987), quantified using Quantus (Promega, Madison, WI, United States), and analyzed on agarose gels to assess the size distribution of DNA fragments. The ideal library size ranged between 350 and 450bp, and DNA samples with average fragments size above these thresholds were sonicated in a Covaris M220 Focused-ultrasonicator (Covaris, Woburn, MA, United States) prior to library preparation.

Dual indexed Illumina DNA libraries were prepared using NEBNext Ultra II DNA Library Prep Kit (New England Biolabs, Ipswich, MA, United States), following the manufacturer’s protocol, with size selection not performed on highly degraded DNA samples. Libraries were amplified with 8–12 PCR cycles, depending on initial starting amount of DNA and later quantified using Quantus; library sizes were assessed using 4200 TapeStation and standard D1000 tapes (Agilent Technologies, Cheadle, United Kingdom). Up to 24 dual-indexed libraries were pooled in equimolar concentration prior to hybridization. Pooled libraries were hybridized with the Angiosperms353 probe set (Johnson et al., 2019; Arbor Biosciences myBaits Target Sequence Capture Kit) following manufacturer’s protocol, ver. 4. Hybridization reactions were performed at 65°C for 24 h, followed by PCR amplification using NEBNext Q5 HotStart HiFi PCR Master Mix (New England BioLabs, Ipswich, MA, United States) and 12 cycles. Final hybridized pools were quantified and profiled as described above for individual libraries. Multiple enriched pools were combined, totaling up to 200 samples per sequencing lane, and sequenced using Illumina HiSeq at Macrogen (Seoul, South Korea). Raw Illumina reads have been deposited in the European Nucleotide Archive (PAFTOL BioProject PRJEB35285 and GAP Bioproject PRJEB49212).

### Bioinformatic and Phylogenomic Analyses

Reads were first cleaned with Trimmomatic to remove barcode and adapter sequences and to remove reads with a quality score below 10 or reads less than 40 bp long (Bolger et al., 2014). Cleaned reads were assembled into genes using the HybPiper assembly pipeline (Johnson et al., 2016) using the expanded Angiosperms353 reference file (McLay et al., 2021). In short, this pipeline first maps reads to the reference file using bwa (Li and Durbin, 2009) and assembles reads into contigs using SPAdes (Bankevich et al., 2012; Prjibelski et al., 2020). Exonerate (Slater and Birney, 2005) is then used to align contigs to the target sequences in the reference file. Exons were then merged with introns to create “supercontigs,” which are exon sequences with flanking introns recovered from the splash zone (Johnson et al., 2016). Supercontigs were created for each gene for each species. Because a whole genome was available for *Ipomoea triloba* (ASM357664v1; Wu et al., 2018), the Angiosperms353 sequences were recovered from the assemblies, using BLAST, as described in Baker et al. (2022). Sequences showing evidence of paralogy were removed from further analysis as has been done in previous phylogenetic analyses using target capture data (Wu et al., 2018; Eserman et al., 2021). There were very few paralog warnings in HybPiper assemblies, an average of 5 of 353 genes per sample, suggesting that paralogy is not a major issue in analyses of Angiosperms353 data in Convolvulaceae. Supercontig alignments for each one of the 353 nuclear loci were generated in PRANK (Löytynoja and Goldman, 2005, 2008; Löytynoja, 2014) and cleaned in Gblocks to remove positions with a gap in greater than 50% of individuals (Castresana, 2000; Talavera and Castresana, 2007). Alignment statistics were calculated with AMAS (Borowiec, 2016). Gblocks filtered alignments were then cleaned to remove samples without a sequence using a custom perl script – batch_Removeblank.pl.^3^

Phylogenetic trees were estimated using maximum likelihood methods in IQ-TREE 2 (Minh et al., 2020) based on a concatenated dataset. Clade support on gene trees was assessed using 1000 ultrafast bootstrap replicates as implemented in IQ-TREE 2 (Hoang et al., 2018). Coalescent analyses were performed in ASTRAL-III using default settings (Zhang et al., 2018), and clade supports were examined using multi-locus bootstrapping (Seo, 2008) and the clade polytomy tests (Sayyari and Mirarab, 2018).

One major goal of this study was to ascertain the placement of *Cuscuta* within the family. Owing to elevated substitution rates associated with photosynthesis loss, parasitic plants can not only be difficult to place in plant phylogeny in their own right, but their inclusion in analyses can also severely affect the resolution and support of other sampled, autotrophic taxa (e.g., Stefanović and Olmstead, 2004). To explore effects of inclusion of *Cuscuta* on our phylogenetic inference, analyses were conducted with two separate datasets: one with 34 species of Convolvulaceae including the functional outgroup *H. madagascariensis*, and the other containing the same taxa with addition of *Cuscuta australis* R. Br.

Alignments, gene trees, and unaligned gene datasets for the three taxon samples (Convolvulaceae and outgroups, Convolvulaceae without *Cuscuta*, and Convolvulaceae with *Cuscuta*) are available at: https://github.com/laeserman/Convolvulaceae_PAFTOL.

## Results

Between 309 and 351 genes of the total 353 genes were recovered for species within Convolvulaceae. Recovery was somewhat lower in outgroup species, with a range of 250-308 genes assembled per species. Within ingroup species, four genes had low recovery with a sequence assembled in only half or fewer of taxa (PAFToL Gene IDs 5354, 6886, 7013, and 7111). The mean sequence length per gene was 739 bp with gene assemblies ranging from 95 to 2796 bp (Supplementary Table 3). Exon alignments for the dataset containing Convolvulaceae and Solanaceae was 683 bp on average and ranged from 75 to 3127 bp in length; alignments contained on average 46% parsimony informative (PI) sites, with a range of 23% to 65%. Supercontig alignments for the dataset containing only Convolvulaceae species including *Cuscuta* were 1827 bp long on average and ranged from 206 to 7810 bp in length; alignments contained on average 25% PI sites, with a range of 13% to 34%. Supercontig alignments for the dataset containing Convolvulaceae without *Cuscuta* were 1774 bp long on average and ranged from 136 to 7567 bp; alignments contained on average 24% PI sites, with a range of 13% to 35% (Supplementary Table 4).

To assess the most suitable outgroup for further analysis of Convolvulaceae, we first included a full dataset of species in Convolvulaceae, sister family to Solanaceae, and more distant outgroup from Montiniaceae. The species tree estimated in ASTRAL-III confirmed the monophyly of the family Convolvulaceae with 100% multilocus bootstrap support. As indicated in previous studies (Stefanović et al., 2002; Stefanović and Olmstead, 2004), *H. madagascariensis* was found here again to be sister to the rest of Convolvulaceae (Supplementary Figure 1). We recognize that in a larger analysis of Angiosperms353 data across all angiosperms, *Cuscuta* was recovered in this position instead of *Humbertia* (treeoflife.kew.org; Baker et al., 2022). It is likely that this broader analysis, using all plant families, was based on comparatively poor alignment due to the high rate of mutation in parasitic plant lineages, resulting in a spurious placement of *Cuscuta*. Because our focused analysis is showing *H. madagascariensis* as sister to the rest of Convolvulaceae, *H. madagascariensis* was used as the functional outgroup in further analyses of family level relationships, allowing for a greater inclusion of captured and assembled genes.

**FIGURE 1:**
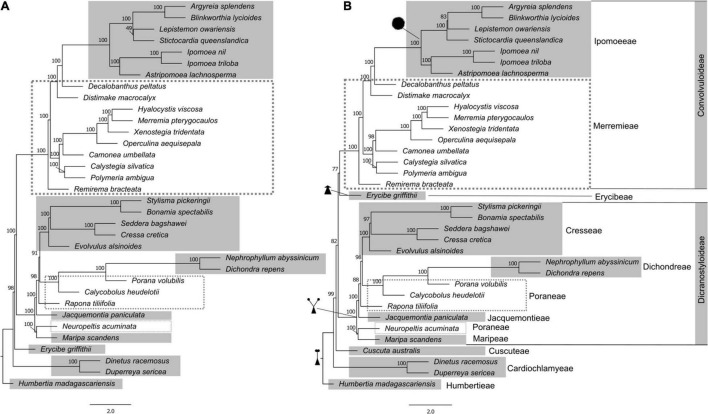
Phylogeny of Convolvulaceae with different dataset composition estimated in ASTRAL-III, exploring the impact of the inclusion of *Cuscuta* in the coalescent analyses. **(A)** without *Cuscuta*; **(B)** with *Cuscuta*. Tribes are indicated, and known synapomorphies for the major clades are illustrated: single style, assumed plesiomorphic for the family; “bifid style”, shared by the Dicranostyloideae clade including tribes Dichondreae, Poraneae, Jacquemontieae, and Maripeae; the lack of a style (sessile stigma) in Erycibeae; and spiny pollen, supporting tribe Ipomoeeae. Node support was assessed by multilocus bootstrapping in ASTRAL-III. ASTRAL-III only estimates internal branch lengths, which are in coalescent units; all terminal branch lengths are set to be equivalent.

With this more inclusive gene dataset, we then assessed relationships in a sample focused only on Convolvulaceae species (Figure 1). Two datasets were generated, one including and another excluding *Cuscuta australis*. The trees resulting from coalescent analysis in ASTRAL-III with and without *Cuscuta* are similar in topology except for the placement of *Erycibe griffithii* C.B.Clarke. When *Cuscuta* is added to the analysis, it is found to have diverged after the diversification of Cardiochlamyeae and is sister to the rest of Convolvulaceae tribes including Ipomoeeae, Merremieae, Erycibeae, Cresseae, Dichondreae, Poraneae, Jacquemontieae, and Maripeae. The placement of *Cuscuta* within the family is supported by 82% multilocus bootstrap replicates in the ASTRAL-III analysis. This placement of *Cuscuta* is the same when the supercontig data are concatenated and analyzed in IQ-TREE 2, except that the support decreases to 59% (Supplementary Figure 2).

## Discussion

The analyses of nuclear genes targeted by the Angiosperms353 universal probes bring a fresh perspective on higher level relationships across Convolvulaceae. Some unanswered evolutionary and systematic questions remain, for which additional sampling will be required. We here provide an overview of the main novelties, hypotheses from previous studies that were confirmed, and outstanding challenges that will need to be addressed in the future.

### Backbone of Convolvulaceae and Its Main Lineages

In most cases, the relationships between the larger clades and tribes are well supported (100% bootstrap) across the phylogenetic tree (Figure 1 and Supplementary Figure 2). The most uncertain is the relationship between Cardiochlamyoideae and the clade that includes Cuscutoideae, Dicranostyloideae, and Convolvuloideae. While this does not impact the classification of the family at higher levels, it will likely hamper evolutionary studies that may aim to investigate processes beyond Dicranostyloideae and Convolvuloideae, and it is therefore an uncertainty that will need to be addressed in future studies, to enable any character evolution, biogeographic, and diversification analyses at the family level.

Humbertioideae and Eryciboideae are both lineages containing a single genus, even a monotypic genus in the case of *Humbertia*. This extraordinary species, a berry-producing tree endemic to Madagascar – *Humbertia madagascariensis* – was found to be sister to the rest of the family Convolvulaceae, as previous results had demonstrated (Stefanović et al., 2003). Placement of *Erycibe* as sister to the clade that contains Convolvuloideae and Dicranostyloideae (Figure 1A, In absence of *Cuscuta*) is also in agreement with earlier analyses; however, its position as sister to Convolvuloideae, in an analysis which includes *Cuscuta* (Figure 1B), is unexpected and without morphological support, to the best of the current knowledge of the family. We hypothesize that this is a methodological artifact, deriving from the addition of a representative of a potentially very long branched lineage (compare phylograms in Supplementary Figure 2).

### Position of *Cuscuta*

The inclusion of *Cuscuta* in Convolvulaceae has, in early systematic studies, been under dispute, with authors having treated it as a separate family (Cuscutaceae) based on its parasitic life form (Dumortier, 1829; Roberty, 1952, 1964; Austin, 1973; Cronquist, 1988; Takhtajan, 1997). Molecular phylogenetic studies targeting the genus have demonstrated that it is, indeed, included in Convolvulaceae (Stefanović et al., 2002; Stefanović and Olmstead, 2004), although its exact position with respect to the remainder of the family has not been established with confidence. In the present study, we confirm the inclusion of *Cuscuta* within Convolvulaceae, with high support (99-100% bootstrap support; Figure 1B and Supplementary Figure 2B). However, the uncertainty of its position within the family remains. The ASTRAL-III analysis shows it as sister to the clade that includes Convolvuloideae, Dicranostyloideae, and *Erycibe*, with a moderate support (82% bootstrap; Figure 1B). This position is novel, and is not one of the potential placements seen in previous analyses (Stefanović and Olmstead, 2004). On the other side, the IQ-TREE 2 analysis of concatenated data places *Cuscuta* as sister to Convolvuloideae and Dicranostyloideae, with exclusion of *Erycibe*, but with weak support only (59% bootstrap; Supplementary Figure 2B). Concatenated data results (Supplementary Figure 2) also point to a likely reason behind the lack of support: a substantial substitution rate elevation and potential long-branch artifacts (Felsenstein, 1978; Bergsten, 2005) observed in many heterotrophic lineages (Nickrent, 2020), including *Cuscuta* (Stefanović and Olmstead, 2004). It is likely that with the addition of further taxon sampling across the family, and, in particular, the inclusion of additional species of *Cuscuta* to span the basal node of this divergent genus, relationships around *Cuscuta* can be resolved with greater support.

The monophyly of Cuscutoideae could also not be tested, because only one representative of this lineage was included in our current study. However, previous molecular phylogenetic analyses, with a broad sampling of ingroup taxa, have strongly demonstrated the monophyly of this subfamily (Stefanović et al., 2002; García et al., 2014). Such scenario is unlikely to change, considering the highly diverging morphology, ecology, and life form of the members of this subfamily, with respect to the rest of the family.

### Monophyly and Circumscription of Dicranostyloideae

Most remarkably, our results offer strong support to the monophyly of one of the largest clades within the family, Dicranostyloideae, the “bifid style” clade. While the monophyly of Convolvuloideae received strong support across the board in previous molecular studies (Stefanović et al., 2002; Stefanović and Olmstead, 2004), this was not the case for Dicranostyloideae, where support varied from weak to moderate. One of the main issues was the unexpected position of *Jacquemontia*. This genus was once placed in tribe Convolvuleae based on morphology, but molecular phylogenetic analyses have suggested, albeit without substantial support, that it would belong in Dicranostyloideae instead (Stefanović et al., 2002; Stefanović and Olmstead, 2004). Our current results not only confirm the placement of *Jacquemontia* within Dicranostyloideae, sister to tribes Cresseae, Poraneae, and Dichondreae, but also suggests this relationship is very strongly supported (100% bootstrap support; Figure 1). Molecular phylogenetic studies by Stefanović et al. (2003) have proposed the circumscription of tribe Cresseae as including the following genera: *Bonamia*, *Cressa* L., *Evolvulus* L., *Hildebrandtia* Vatke (including *Cladostigma* Radlk. and *Sabaudiella* Chiov.), *Itzaea* Standl. & Steyerm., *Neuropeltis* Wall., *Neuropeltopsis* Ooststr., *Seddera* Hochst., *Stylisma* Raf., and *Wilsonia* G.L.Chu. Our phylogenetic analyses demonstrate with confidence that this tribe is not monophyletic in its current circumscription, due to the position of *Neuropeltis*, which is placed outside of any of the clades that include Poraneae, and sister to *Maripa* Aubl. In previous analyses, it was resolved as sister to the clade that included *Bonamia, Itzaea, Calycobolus, Dipteropeltis*, and *Rapona*, albeit with weak support (Stefanović et al., 2002). Poraneae, in its turn, are demonstrated to be paraphyletic, with *Rapona* Baill. being sister to the clade that includes Cresseae, Dichondreae, and part of Poraneae (*Porana* Burm. f. and *Calycobolus*).

Taken together, these are completely new insights into the relationships within Dicranostyloideae and these findings reinforce the importance of additional data in improving the analyses. Hence, these results are far from definitive, considering that at least ten genera of Dicranostyloideae still remain to be sampled, which could be key in clarifying at last the tribal delimitation within this clade.

### Non-monophyly of “Merremieae” and Shifting Relationships to Ipomoeeae

Our analyses also confirm with great confidence the non-monophyly of the already dissolved “Merremieae”, whose genera have been classified as “incertae sedis” (i.e., without tribal placement) due to uncertainty regarding the delimitation of this tribe (Stefanović et al., 2003). The molecular phylogenetic analyses of Simões et al. (2015), which targeted this complex group in detail, suggested for the first time the placement of *Daustinia, Merremia*, and *Decalobanthus* within the clade that contains tribe Ipomoeeae with maximum support, as a grade, with each genus forming its own separate clade. The remainder of the tribe was resolved across multiple lineages, with a main larger clade that contained most of the genera of the “tribe” - *Distimake, Operculina* Silva Manso, *Camonea* Raf., *Hewittia* Wight & Arn., *Hyalocystis* Hallier f., *Xenostegia* D.F. Austin & Staples – and a number of unplaced species of *Merremia*. However, these results were generally doubtful, because most of the relationships were not significantly supported, which was attributed to the need of additional sequence data.

With our current data (Figure 1 and Supplementary Figure 2), we have obtained a similar tree topology to that recovered previously for this group (Simões et al., 2015), but this time around the non-monophyly of “Merremieae” is for the first time demonstrated with high support. In addition, one completely novel relationship is presented: the largest genus previously segregated from *Merremia* s.l. – *Distimake* Raf. – is now actually found to be sister to the clade that includes *Decalobanthus* and Ipomoeeae (100% bootstrap).

### Convolvuleae Nested Within “Merremieae”

An additional novel finding is the position of tribe Convolvuleeae within the “Merremieae”, as sister to the largest “merremioid” clade, with strong support (100% bootstrap). Of all genera of Convolvuleae, *Convolvulus* was not sampled for our supercontig analysis, but was sampled for the exon analysis (Supplementary Figure 1), which supports its placement within the Convolvuleae and sister to *Calystegia*, consistent with prior results (Williams et al., 2014). This finding further supports the need to densely sample and reassess the tribal placement of the genera formerly included in “Merremieae”, and their potential segregation into multiple monophyletic tribes.

## Conclusion

In the history of classifications of Convolvulaceae, molecular phylogenetic analyses have been paramount to elucidate relationships within the family, where morphology was conflicting. As a result, new characters are arising as potentially taxonomically informative, and a new path for evolutionary and biogeographic hypotheses for the family is being illuminated.

The molecular phylogeny of Convolvulaceae by Stefanović et al. (2002) remains the most taxonomically comprehensive thus far. However, lack of support at the deeper relationships within the family hindered progress in systematics and evolutionary studies at higher taxonomic levels (subfamilies and tribes). Subsequent molecular phylogenetic studies have focused on smaller taxonomic groups within the family, and while informative, they have missed the taxonomic breadth that is necessary to fully resolve some of the outstanding problems within the family. A new age of genomic data, and the rise of large-scale collaborative projects that are globally fast-forwarding the sequencing of plant species, have provided an extraordinary amount of data, which we have accessed in this study as means to explore the deeper conflicts in the phylogeny of Convolvulaceae. The major clades within the family obtained in earlier phylogenetic studies seem strongly corroborated by the present analyses, although the tribal delimitation is still problematic due to the uncertainty of the classification of the “Merremieae” and the tribes within the Dicranostyloideae clade. The monophyly of several genera is also still to be further investigated (e.g., *Jacquemontia*, *Bonamia, Calycobolus*), which re-circumscription is likely to also have an impact in the tribal delimitation. While some key higher-level relationships are for the first time here consolidated, it is clear that a top-down reclassification of the family can now only be possible once this phylogenomic approach is expanded to additional taxa, with deeper sampling at generic and species level. Addition of key taxa in Convolvulaceae may lead to substantial taxonomic changes, paralleling those observed in Apiaceae (Clarkson et al., 2021) and Commelinaceae (Zuntini et al., 2021).

## Data Availability Statement

The datasets presented in this study can be found in online repositories. Raw Illumina reads can be found in the European Nucleotide Archive (PAFTOL BioProject: PRJEB35285 and GAP Bioproject: PRJEB49212). Alignments, gene trees, and unaligned gene datasets are available at: https://github.com/laeserman/Convolvulaceae_PAFTOL. The names of the repository/repositories and accession number(s) can be found in the article Supplementary Material.

## Author Contributions

AS coordinated the study and the writing of the manuscript, with collaboration of all co-authors. LE performed the analyses and contributed to manuscript writing. AZ, LC, and TU provided significant scientific contributions to the discussions and manuscript writing. OM and SRoy performed the laboratorial work. SRok collected samples. WB and FF conceived and supervised the generation of the genetic data as part of PAFToL project and contributed scientifically to the discussions and manuscript preparation. SS supervised the analyses, the scientific discussions, and the preparation of the manuscript. All authors contributed to the article and approved the submitted version.

## Conflict of Interest

The authors declare that the research was conducted in the absence of any commercial or financial relationships that could be construed as a potential conflict of interest.

## Publisher’s Note

All claims expressed in this article are solely those of the authors and do not necessarily represent those of their affiliated organizations, or those of the publisher, the editors and the reviewers. Any product that may be evaluated in this article, or claim that may be made by its manufacturer, is not guaranteed or endorsed by the publisher.

## Footnotes

1
https://treeoflife.kew.org

2
https://www.genomicsforaustralianplants.com/

3
https://github.com/laeserman/Convolvulaceae_PAFTOL

## Supplementary Material

Supplementary Figure 1 Phylogeny of Convolvulaceae including outgroups from Solanaceae and Montiniaceae. **(A)** Tree estimated from a concatenated dataset of 349 genes analyzed in IQ-TREE2 with 1000 ultrafast bootstrap replicates. **(B)** Tree estimated in ASTRAL-III using gene trees from 349 gene trees. The main result from this analysis is that Convolvulaceae is monophyletic with 100% bootstrap support. Additionally, *Humbertia madagascariensis* is sister to the rest of the Convolvulaceae. Further analyses presented here use *H. madagascariensis* as the functional outgroup to improve alignment and thus tree estimation.

Supplementary Figure 2 Phylogeny of Convolvulaceae with different dataset composition, exploring the impact of the inclusion of *Cuscuta* in the analyses. **(A)** without *Cuscuta*; **(B)** with *Cuscuta*. Both trees were estimated using a concatenated dataset of 349 genes that were aligned in PRANK and cleaned in Gblocks. The concatenated dataset was analyzed in IQ-TREE2 with 1000 ultrafast bootstrap replicates.

## References

[B1] Austin D. F. (1973). The American Erycibeae (Convolvulaceae): *Capitalize Maripa*, *Dicranostyles*, and *Lysiostyles* I. Systematics. *Ann. Mo. Bot. Gard.* 60 306–412. 10.2307/2395089

[B2] Backlund A. Bremer K. (1998). To Be or Not to Be. Principles of Classification and Monotypic Plant Families. *Taxon* 47 391–400. 10.2307/1223768

[B3] Baker W. J. Bailey P. Barber V. Barker A. Bellot S. Bishop D. (2022). A Comprehensive Phylogenomic Platform for Exploring the Angiosperm Tree of Life. *Syst. Biol.* 71 301–319. 10.1093/sysbio/syab035 33983440 PMC8830076

[B4] Baker W. J. Dodsworth S. Forest F. Graham S. W. Johnson M. G. McDonnell A. (2021). Exploring Angiosperms353: an open, community toolkit for collaborative phylogenomic research on flowering plants. *Am. J. Bot.* 108 1059–1065. 10.1002/ajb2.1703 34293179

[B5] Banerjee A. Stefanović S. (2019). Caught in action: fine-scale plastome evolution in the parasitic plants of *Cuscuta* section *Ceratophorae* (Convolvulaceae). *Plant Mol. Biol.* 100 621–634. 10.1007/s11103-019-00884-0 31140020

[B6] Banerjee A. Stefanović S. (2020). Reconstructing plastome evolution across the phylogenetic backbone of the parasitic plant genus *Cuscuta* (Convolvulaceae). *Bot. J. Linn. Soc.* 194 423–438. 10.1093/botlinnean/boaa056

[B7] Bankevich A. Nurk S. Antipov D. Gurevich A. A. Dvorkin M. Kulikov A. S. (2012). SPAdes: a new genome assembly algorithm and its applications to single-cell sequencing. *J. Comp. Biol.* 19 455–477. 10.1089/cmb.2012.0021 22506599 PMC3342519

[B8] Beaulieu W. T. Panaccione D. G. Quach Q. N. Smoot K. L. Clay K. (2021). Diversification of ergot alkaloids and heritable fungal symbionts in morning glories. *Commun. Biol.* 4:1362. 10.1038/s42003-021-02870-z 34873267 PMC8648897

[B9] Bergsten J. (2005). A review of long-branch attraction. *Cladistics* 21 163–193. 10.1111/j.1096-0031.2005.00059.x 34892859

[B10] Bolger A. M. Lohse M. Usadel B. (2014). Trimmomatic: a flexible trimmer for Illumina sequence data. *Bioinformatics* 30 2114–2120. 10.1093/bioinformatics/btu170 24695404 PMC4103590

[B11] Borowiec M. L. (2016). AMAS: a fast tool for alignment manipulation and computing of summary statistics. *PeerJ* 4:e1660. 10.7717/peerj.1660 26835189 PMC4734057

[B12] Braukmann T. W. Kuzmina M. Stefanović S. (2013). Plastid genome evolution across the genus *Cuscuta* (Convolvulaceae): two clades within subgenus *Grammica* exhibit extensive gene loss. *J. Exp. Bot.* 64 977–989. 10.1093/jxb/ers391 23349139 PMC3580819

[B13] Brewer G. E. Clarkson J. J. Maurin O. Zuntini A. R. Barber V. Bellot S. (2019). Factors Affecting Targeted Sequencing of 353 Nuclear Genes From Herbarium Specimens Spanning the Diversity of Angiosperms. *Front. Plant Sci.* 10:1102. 10.3389/fpls.2019.01102 31620145 PMC6759688

[B14] Buril M. T. Simões A. R. Carine M. Alves M. (2013). *Austinia*, a new genus of Convolvulaceae from Brazil. *Phytotaxa* 186 254–260. 10.11646/phytotaxa.186.5.2

[B15] Buril M. T. Simões A. R. Carine M. Alves M. (2015). *Daustinia*, a replacement name for *Austinia* (Convolvulaceae). *Phytotaxa* 197:60. 10.11646/phytotaxa.197.1.8

[B16] Carruthers T. Muñoz-Rodríguez P. Wood J. R. I. Scotland R. W. (2020). The temporal dynamics of evolutionary diversification in Ipomoea. *Mol. Phylogenet. Evol.* 146:106768. 10.1016/j.ympev.2020.106768 32081764

[B17] Castresana J. (2000). Selection of conserved blocks from multiple alignments for their use in phylogenetic analysis. *Mol. Biol. Evol.* 17 540–552. 10.1093/oxfordjournals.molbev.a026334 10742046

[B18] Clarkson J. J. Zuntini A. R. Maurin O. Downie S. R. Plunkett G. M. Nicolas A. N. (2021). A higher-level nuclear phylogenomic study of the carrot family (Apiaceae). *Am. J. Bot.* 108 1252–1269. 10.1002/ajb2.1701 34287829

[B19] Cronquist A. (1988). *The Evolution And Classification Of Flowering Plants.* Bronx: The New York Botanical Garden.

[B20] Dodsworth S. Pokorny L. Johnson M. G. Kim J. T. Maurin O. Wickett N. J. (2019). Hyb-Seq for Flowering Plant Systematics. *Trends in Plant Sci.* 24 887–891. 10.1016/j.tplants.2019.07.011 31477409

[B21] Doyle J. J. Doyle J. L. (1987). A rapid DNA isolation procedure from small quantities of fresh leaf tissue. *Phytochem. Bull.* 19 11–15.

[B22] Dumortier B.-C. (1829). *Analyse Des Plantes.* Paris: Tournay.

[B23] Eich E. (2008). *Solanaceae and Convolvulaceae: Secondary Metabolites; Biosynthesis, Chemotaxonomy, Biological and Economic Significance; A Handbook.* Berlin: Springer. 10.1007/978-3-540-74541-9

[B24] Eserman L. A. Thomas S. K. Coffey E. E. D. Leebens-Mack J. H. (2021). Target sequence capture in orchids: developing a kit to sequence hundreds of single-copy loci. *Appl. Plant Sci.* 9:11416. 10.1002/aps3.11416 34336404 PMC8312744

[B25] Eserman L. A. Tiley G. P. Jarret R. L. Leebens-Mack J. H. Miller R. E. (2014). Phylogenetics and diversification of morning glories (tribe Ipomoeeae, Convolvulaceae) based on whole plastome sequences. *Am. J. Bot.* 101 92–103. 10.3732/ajb.1300207 24375828

[B26] Felsenstein J. (1978). Cases in which parsimony or compatibility methods will be positively misleading. *Syst. Zool.* 27 401–410. 10.2307/2412923

[B27] García M. A. Costea M. Kuzmina M. Stefanović S. (2014). Phylogeny, character evolution, and biogeography of *Cuscuta* (dodders; Convolvulaceae) inferred from coding plastid and nuclear sequences. *Am. J. Bot.* 101 670–690. 24688058 10.3732/ajb.1300449

[B28] Hoang D. T. Chernomor O. von Haeseler A. Minh B. Q. Vinh L. S. (2018). UFBoot2: improving the ultrafast bootstrap approximation. *Mol. Biol. Evol.* 35 518–522. 10.1093/molbev/msx281 29077904 PMC5850222

[B29] Ibiapino A. García M. A. Amorim B. Baez M. A. Costea M. Stefanović S. (2022). The evolution of cytogenetic traits in *Cuscuta* (Convolvulaceae), the genus with the most diverse chromosomes in angiosperms. *Front. Plant. Sci.* 13:842260. 10.3389/fpls.2022.842260 35432411 PMC9011109

[B30] Johnson M. G. Gardner E. M. Liu Y. Medina R. Goffinet B. Shaw A. J. (2016). HybPiper: extracting coding sequence and introns for phylogenetics from high-throughput sequencing reads using target enrichment. *Appl. Plant Sci.* 4:apps.1600016. 10.3732/apps.1600016 27437175 PMC4948903

[B31] Johnson M. G. Pokorny L. Dodsworth S. Botigué L. R. Cowan R. S. Devault A. (2019). A Universal Probe Set for Targeted Sequencing of 353 Nuclear Genes from Any Flowering Plant Designed Using k-Medoids Clustering. *Syst. Biol.* 68 594–606. 10.1093/sysbio/syy086 30535394 PMC6568016

[B32] Larridon I. Zuntini A. R. Léveillé-Bourret E. Barrett R. L. Starr J. R. Muasya A. M. (2021). A new classification of Cyperaceae (Poales) supported by phylogenomic data. *J. Syst. Evol.* 59 852–895. 10.1111/jse.12757

[B33] Lee A. K. Gilman I. S. Srivastav M. Lerner A. D. Donoghue M. J. Clement W. L. (2021). Reconstructing Dipsacales phylogeny using Angiosperms353: issues and insights. *Am. J. Bot.* 108 1122–1142. 10.1002/ajb2.1695 34254290 PMC8362060

[B34] Li H. Durbin R. (2009). Fast and accurate short read alignment with Burrows-Wheeler transform. *Bioinformatics* 25 1754–1760. 10.1093/bioinformatics/btp324 19451168 PMC2705234

[B35] Lin Y. Li P. Zhang Y. Akhter D. Pan R. Fu Z. (2022). Unprecedented organelle genomic variation in morning glories reveal independent evolutionary scenario of parasitic plants and the diversification of plant mitochondrial complexes. *BMC Biol.* 20:49. 10.1186/s12915-022-01250-1 35172831 PMC8851834

[B36] Löytynoja A. (2014). “Phylogeny-aware alignment with PRANK,” in *Multiple Sequence Alignment Methods*, ed. Russell D. J. (Totowa: Humana Press), 155–170. 10.1007/978-1-62703-646-7_10 24170401

[B37] Löytynoja A. Goldman N. (2005). An algorithm for progressive multiple alignment of sequences with insertions. *Proc. Natl. Acad. Sci. U.S.A.* 102 10557–10562. 10.1073/pnas.0409137102 16000407 PMC1180752

[B38] Löytynoja A. Goldman N. (2008). Phylogeny-aware gap placement prevents errors in sequence alignment and evolutionary analysis. *Science* 320 1632–1635. 10.1126/science.1158395 18566285

[B39] McLay T. G. B. Birch J. L. Gunn B. F. Ning W. Tate J. A. Nauheimer L. (2021). New targets acquired: improving locus recovery from the Angiosperms353 probe set. *Appl. Plant Sci.* 9:e11420. 10.1002/aps3.11420 34336399 PMC8312740

[B40] Minh B. Q. Schmidt H. A. Chernomor O. Schrempf D. Woodhams M. D. von Haeseler A. (2020). IQ-TREE 2: new models and efficient methods for phylogenetic inference in the genomic era. *Mol. Biol. Evol.* 37 1530–1534. 10.1093/molbev/msaa015 32011700 PMC7182206

[B41] Muñoz-Rodríguez P. Carruthers T. Wood J. R. I. Williams B. R. M. Weitemier K. Kronmiller B. (2019). A taxonomic monograph of *Ipomoea* integrated across phylogenetic scales. *Nat. Plants* 5 1136–1144. 10.1038/s41477-019-0535-4 31712754

[B42] Nickrent D. L. (2020). Parasitic angiosperms: how often and how many? *Taxon* 69 5–27. 10.1002/tax.12195

[B43] One Thousand Plant Transcriptomes Initiative (2019). One thousand plant transcriptomes and the phylogenomics of green plants. *Nature* 574 679–685. 10.1038/s41586-019-1693-2 31645766 PMC6872490

[B44] Pérez-Escobar O. A. Dodsworth S. Bogarín D. Bellot S. Balbuena J. A. Schley R. J. (2021). Hundreds of nuclear and plastid loci yield novel insights into orchid relationships. *Am. J. Bot.* 108 1166–1180. 10.1002/ajb2.1702 34250591

[B45] Petrongari F. P. Simões A. R. Simão-Bianchini R. (2018). New combinations and lectotypifications in *Distimake* Raf. (Convolvulaceae). *Phytotaxa* 340 297–300. 10.11646/phytotaxa.340.3.12

[B46] POWO (2022). *Plants of the World Online.* London UK: Royal Botanic Gardens, Kew,

[B47] Prjibelski A. Antipov D. Meleshko D. Lapidus A. Korobeynikov A. (2020). Using SPAdes de novo assembler. *Curr. Protoc. Bioinform.* 70:e102. 10.1002/cpbi.102 32559359

[B48] Roberty G. (1952). Genera Convolvulacearum. *Candollea* 14 11–60.

[B49] Roberty G. (1964). Les genres des Convolvulacées (esquisse). *Boissiera* 10 129–156.

[B50] Sayyari E. Mirarab S. (2018). Testing for polytomies in phylogenetic species trees using quartet frequencies. *Genes* 9:132. 10.3390/genes9030132 29495636 PMC5867853

[B51] Seo T. K. (2008). Calculating bootstrap probabilities of phylogeny using multilocus sequence data. *Mol. Biol. Evol.* 25 960–971. 10.1093/molbev/msn043 18281270

[B52] Shee Z. Q. Frodin D. G. Cámara-Leret R. Pokorny L. (2020). Reconstructing the Complex Evolutionary History of the Papuasian Schefflera Radiation Through Herbariomics. *Front. Plant Sci.* 11:258. 10.3389/fpls.2020.00258 32265950 PMC7099051

[B53] Simões A. R. More S. (2018). Synopsis and lectotypification of *Distimake rhyncorhiza* (Dalzell) Simões & Staples (Convolvulaceae): a little known species from the Western Ghats (India). *Phytotaxa* 336 293–298. 10.11646/phytotaxa.336.3.8

[B54] Simões A. R. Staples G. W. (2017). Dissolution of tribe Merremieae (Convolvulaceae) and a classification for its constituent genera. *Bot. J. Linn. Soc.* 183 561–586. 10.1093/botlinnean/box007

[B55] Simões A. R. Culham A. Carine M. (2015). Resolving the unresolved tribe: a molecular phylogenetic framework for Merremieae (Convolvulaceae). *Bot. J. Linn. Soc.* 179 374–387. 10.1111/boj.12339

[B56] Simões A. R. Pisuttimarn P. Pornpongrungrueng P. Chatrou L. W. (2020). New combinations in *Decalobanthus* (Convolvulaceae). *Kew Bull.* 75:55. 10.1007/s12225-020-09907-2

[B57] Slater G. S. Birney E. (2005). Automated generation of heuristics for biological sequence comparison. *BMC Bioinform.* 6:31. 10.1186/1471-2105-6-31 15713233 PMC553969

[B58] Slimp M. Williams L. D. Hale H. Johnson M. G. (2021). On the potential of Angiosperms353 for population genomic studies. *Appl. Plant Sci.* 9:e11419. 10.1002/aps3.11419 34336401 PMC8312745

[B59] Staples G. W. (2006). Revision of Asiatic Poraneae (Convolvulaceae) - *Cordisepalum*, *Dinetus*, *Duperreya*, *Porana*, *Poranopsis* and *Tridynamia*. *Blumea* 51 403–491. 10.3767/000651906X622067

[B60] Staples G. W. Brummitt R. K. (2007). “Convolvulaceae,” in *Flowering Plants of the World*, eds Heywood V. H. Brummitt R. K. Culham A. Seberg O. (Richmond Hill: Firefly Books), 108–110.

[B61] Staples G. W. Simões A. R. Austin D. F. (2020). A Monograph of Operculina (Convolvulaceae). *Ann. Mo. Bot. Gard.* 105 64–138. 10.3417/2020435 2020435

[B62] Stefanović S. Olmstead R. G. (2004). Testing the Phylogenetic Position of a Parasitic Plant (Cuscuta, Convolvulaceae, Asteridae): bayesian Inference and the Parametric Bootstrap on Data Drawn from Three Genomes. *Syst. Biol.* 53 384–399. 10.1080/10635150490445896 15503669

[B63] Stefanović S. Austin D. F. Olmstead R. G. (2003). Classification of Convolvulaceae: a phylogenetic approach. *Syst. Bot.* 28 791–806.

[B64] Stefanović S. Krueger L. Olmstead R. G. (2002). Monophyly of the Convolvulaceae and circumscription of their major lineages based on DNA sequences of multiple chloroplast loci. *Am. J. Bot.* 89 1510–1522. 10.3732/ajb.89.9.1510 21665753

[B65] Takhtajan A. (1997). *Diversity And Classification Of Flowering Plants.* New York, NY: Columbia University Press.

[B66] Talavera G. Castresana J. (2007). Improvement of phylogenies after removing divergent and ambiguously aligned blocks from protein sequence alignments. *Syst. Biol.* 56 564–577. 10.1080/10635150701472164 17654362

[B67] Thomas S. K. Liu X. Du Z.-Y. Dong Y. Cummings A. Pokorny L. (2021). Comprehending Cornales: phylogenetic reconstruction of the order using the Angiosperms353 probe set. *Am. J. Bot.* 108 1112–1121. 10.1002/ajb2.1696 34263456 PMC8361741

[B68] Williams B. R. Mitchell T. C. Wood J. R. I. Harris D. J. Scotland R. W. Carine M. A. (2014). Integrating DNA barcode data in a monographic study of *Convolvulus*. *Taxon* 63 1287–1306. 10.12705/636.9

[B69] Wu S. Lau K. H. Cao Q. Hamilton J. P. Sun H. Zhou C. (2018). Genome sequences of two diploid wild relatives of cultivated sweetpotato reveal targets for genetic improvement. *Nat. Commun.* 9:4580. 10.1038/s41467-018-06983-8 30389915 PMC6214957

[B70] Zhang C. Rabiee M. Sayyari E. Mirarab S. (2018). ASTRAL-III: polynomial time species tree reconstruction from partially resolved gene trees. *BMC Bioinform.* 19:153. 10.1186/s12859-018-2129-y 29745866 PMC5998893

[B71] Zuntini A. R. Frankel L. P. Pokorny L. Forest F. Baker W. J. (2021). A comprehensive phylogenomic study of the monocot order Commelinales, with a new classification of Commelinaceae. *Am. J. Bot.* 108 1066–1086. 10.1002/ajb2.1698 34278560

