# Supplementary figures and images for: A Bird’s Eye View of the Systematics of Convolvulaceae: Novel Insights From Nuclear Genomic Data
Source: Front Plant Sci. 2022 Jul 14;13:889988. doi: 10.3389/fpls.2022.889988 (PMC9331175; doi:10.3389/fpls.2022.889988)

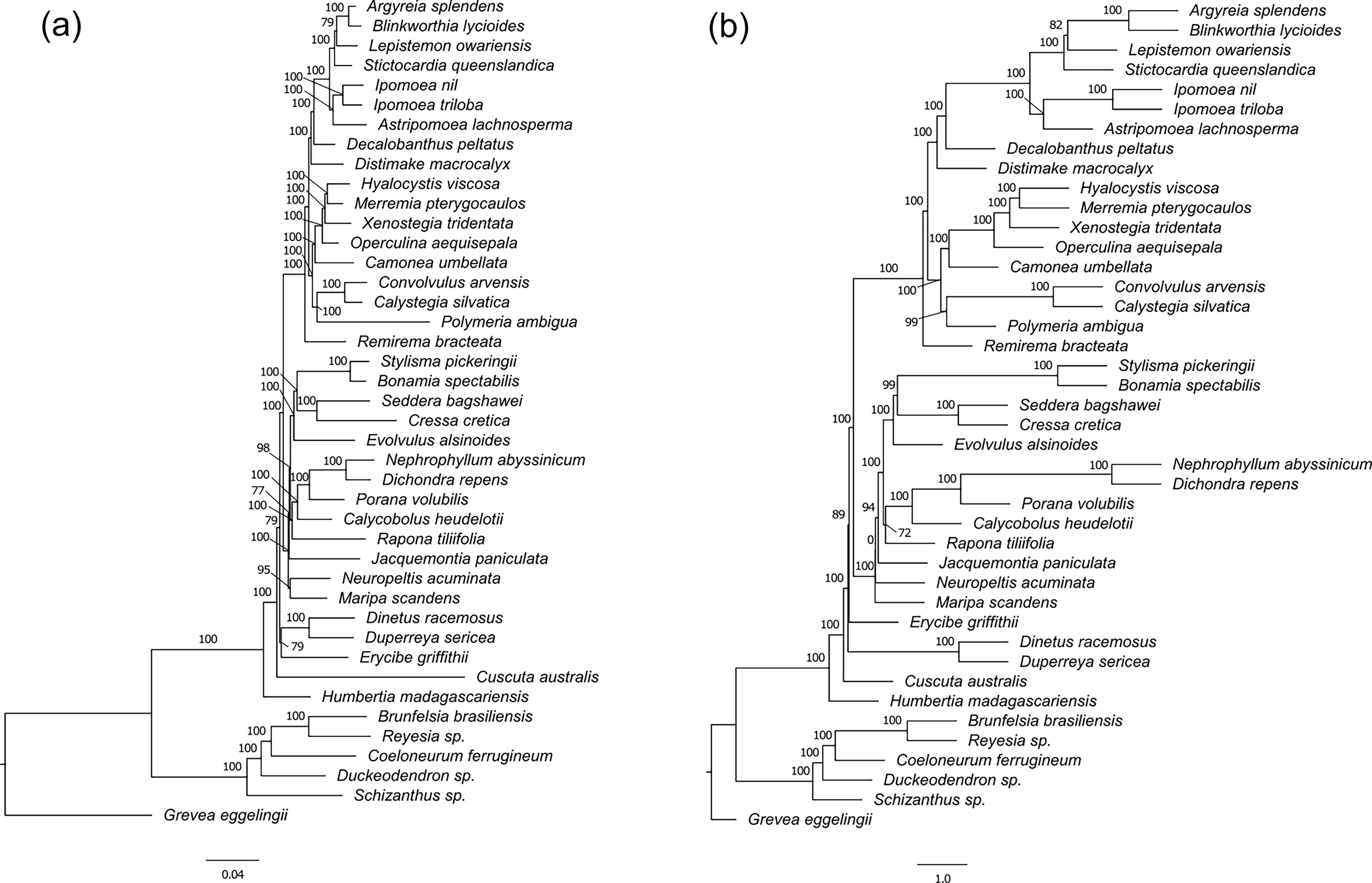

Supplement: Supplementary Figure 1 — Phylogeny of Convolvulaceae including outgroups from Solanaceae and Montiniaceae. (A) Tree estimated from a concatenated dataset of 349 genes analyzed in IQ-TREE2 with 1000 ultrafast bootstrap replicates. (B) Tree estimated in ASTRAL-III using gene trees from 349 gene trees. The main result from this analysis is that Convolvulaceae is monophyletic with 100% bootstrap support. Additionally, Humbertia madagascariensis is sister to the rest of the Convolvulaceae. Further analyses presented here use H. madagascariensis as the functional outgroup to improve alignment and thus tree estimation. [file Image_1.PNG]

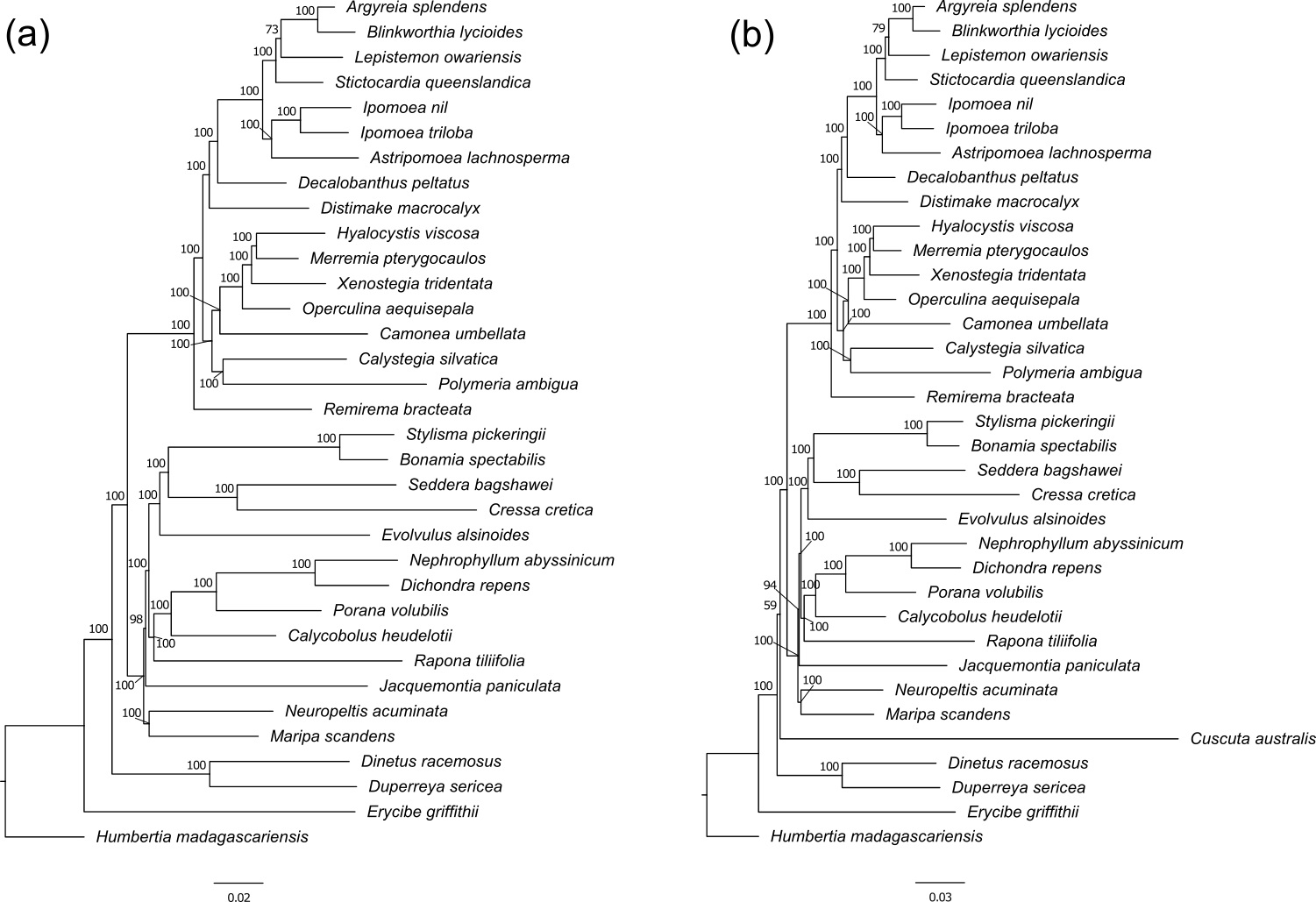

Supplement: Supplementary Figure 2 — Phylogeny of Convolvulaceae with different dataset composition, exploring the impact of the inclusion of Cuscuta in the analyses. (A) without Cuscuta; (B) with Cuscuta. Both trees were estimated using a concatenated dataset of 349 genes that were aligned in PRANK and cleaned in Gblocks. The concatenated dataset was analyzed in IQ-TREE2 with 1000 ultrafast bootstrap replicates. [file Image_2.JPEG]
