# Supplementary material for: A Bird’s Eye View of the Systematics of Convolvulaceae: Novel Insights From Nuclear Genomic Data
Source: Front Plant Sci. 2022 Jul 14;13:889988. doi: 10.3389/fpls.2022.889988 (PMC9331175; doi:10.3389/fpls.2022.889988)
Supplement: Supplementary file 3 [file Table_1.docx]

**Supplementary Table 1**

List of accepted genera (POWO 2022) and their tribal placement under the current classification (sensu Stefanović et al. 2003); number of species updated according to specialised literature (e.g., Petrongari et al. 2018, Simões & More 2018, Simões et al. 2020, Staples et al. 2020).

| **Tribe** | **No. of species** |  | | |
| --- | --- | --- | --- | --- |
| **Cuscuteae** | | | |  |
| *Cuscuta* | 218 | |  |  |
| **Humbertieae** | | | |  |
| *Humbertia* | 1 | |  |  |
| **Erycibeae** | | | |  |
| *Erycibe* | 69 | |  |  |
| **Cardiochlamyeae** | | | |  |
| *Cardiochlamys* | 2 | |  |  |
| *Cordisepalum* | 2 | |  |  |
| *Dinetus* | 7 | |  |  |
| *Duperreya* | 3 | |  |  |
| *Porana* | 2 | |  |  |
| *Poranopsis* | 4 | |  |  |
| *Tridynamia* | 1 | |  |  |
| **Cresseae** |  | |  |  |
| *Bonamia* | 68 | |  |  |
|  |  | |  |  |
| *Cladostigma* | 3 | |  |  |
| *Cressa* | 4 | |  |  |
| *Evolvulus* | 102 | |  |  |
| *Hildebrandtia* | 11 | |  |  |
| *Itzaea* | 1 | |  |  |
| **Maripae** |  | |  |  |
| *Lysiostyles* | 1 | |  |  |
| *Maripa* | 19 | |  |  |
| *Dicranostyles* | 14 | |  |  |
| **Dichondreae** |  | |  |  |
| *Dichondra* | 15 | |  |  |
| *Falkia* | 3 | |  |  |
| *Nephrophyllum* | 1 | |  |  |
| *Petrogenia* | 1 | |  |  |
| *Dipteropeltis* | 3 | |  |  |
| *Metaporana* | 6 | |  |  |
| *Rapona* | 1 | |  |  |
| *Calycobolus* | 24 | |  |  |
| **Cresseae** |  | |  |  |
| *Neuropeltis* | 12 | |  |  |
| *Neuropeltopsis* | 1 | |  |  |
| *Seddera* | 27 | |  |  |
| *Stylisma* | 6 | |  |  |
| *Wilsonia* | 3 | |  |  |
| **Jacquemontieae** |  | |  |  |
| *Jacquemontia* | 108 | |  |  |
| **Ipomoeeae** |  | |  |  |
| *Argyreia* | 136 | |  |  |
| *Astripomoea* | 11 | |  |  |
| *Blinkworthia* | 2 | |  |  |
| *Ipomoea* | 659 | |  |  |
| *Lepistemon* | 7 | |  |  |
| *Lepistemonopsis* | 1 | |  |  |
| *Paralepistemon* | 2 | |  |  |
| *Rivea* | 3 | |  |  |
| *Stictocardia* | 11 | |  |  |
| *Turbina* | 1 | |  |  |
| **“Merremieae”** |  | |  |  |
| *Camonea* | 4 | |  |  |
| *Daustinia* | 1 | |  |  |
| *Decalobanthus* | 17 | |  |  |
| *Distimake* | 47 | |  |  |
| *Hewittia* | 1 | |  |  |
| *Hyalocystis* | 2 | |  |  |
| *Merremia* | 57 | |  |  |
| *Remirema* | 1 | |  |  |
| *Operculina* | 14 | |  |  |
| *Xenostegia* | 7 | |  |  |
| **Aniseieae** |  | |  |  |
| *Aniseia* | 3 | |  |  |
| *Iseia* | 1 | |  |  |
| *Odonellia* | 2 | |  |  |
| *Tetralocularia* | 1 | |  |  |
| **Convolvuleae** |  | |  |  |
| *Convolvulus* | 206 | |  |  |
| *Calystegia* | 27 | |  |  |
| *Polymeria* | 9 | |  |  |
