# Supplementary material for: A Bird’s Eye View of the Systematics of Convolvulaceae: Novel Insights From Nuclear Genomic Data
Source: Front Plant Sci. 2022 Jul 14;13:889988. doi: 10.3389/fpls.2022.889988 (PMC9331175; doi:10.3389/fpls.2022.889988)
Supplement: Supplementary file 4 [file Table_2.docx]

**Supplementary Table 2.** Individuals sampled for phylogenomic analysis of the family Convolvulaceae. Species in the Montiniaceae and Solanaceae were used only for outgroup selection.

| **Data Source*** | **Family** | **Genus** | **Species** | **Voucher** | **Accession** | **No. of Genes** |
| --- | --- | --- | --- | --- | --- | --- |
| PAFTOL | Convolvulaceae | Argyreia | splendens | Chase, M.W. 3836 (K) | ERR7621886 | 341 |
| PAFTOL | Convolvulaceae | Astripomoea | lachnosperma | Heath, A.; Heath, R. 602 (K) | ERR7621763 | 344 |
| PAFTOL | Convolvulaceae | Blinkworthia | lycioides | Larsen, K.; Larsen, S.S. 34012 (K) | ERR7621763 | 318 |
| PAFTOL | Convolvulaceae | Bonamia | spectabilis | Chase, M.W. 3893 (K) | ERR7621887 | 345 |
| PAFTOL | Convolvulaceae | Calycobolus | heudelotii | s.n. 7 (K) | TBC | 349 |
| GAP | Convolvulaceae | Calystegia | silvatica | Messina, A. 1240 (K) | ERR7621887 | 344 |
| PAFTOL | Convolvulaceae | Camonea | umbellata | Eanghourt, K.; Sophal, M.; Kosal, S. 177 (K) | TBC | 323 |
| PAFTOL | Convolvulaceae | Cressa | cretica | Mashaly, I.; Boulos, L. 20246 (K) | ERR7621775 | 333 |
| PAFTOL | Convolvulaceae | Cuscuta | australis | Leon, C.J.; Lin Y-L. 860 (K) | ERR7619223 | 329 |
| PAFTOL | Convolvulaceae | Decalobanthus | peltatus | Pooma, R.; Pattharahirantricin, N.; Sirimongkol, S. 6529 (K) | TBC | 340 |
| PAFTOL | Convolvulaceae | Dichondra | repens | Borhidi, A.; Iversen, S.T.; Mziray, W.R. 85147 (K) | ERR7621802 | 344 |
| PAFTOL | Convolvulaceae | Dinetus | racemosus | Sino-British Plant Exped. Team 14 (K) | TBC | 342 |
| PAFTOL | Convolvulaceae | Distimake | macrocalyx | Perdiz, R.O.; Souza, S.S.; Santos, V.S. 1188 (K) | TBC | 348 |
| GAP | Convolvulaceae | Duperreya | sericea | Purdie, R.W. 7328 (K) | ERR7599419 | 350 |
| PAFTOL | Convolvulaceae | Erycibe | griffithii | Gardette, E. EG 2250 (K) | ERR7621790 | 309 |
| GAP | Convolvulaceae | Evolvulus | alsinoides | N.R. Neagle; H. Stewart BS612-868 (K) | ERR7599763 | 343 |
| PAFTOL | Convolvulaceae | Humbertia | madagascariensis | Razakamalala, R. 4045 (K) | ERR7621788 | 341 |
| PAFTOL | Convolvulaceae | Hyalocystis | viscosa | Wieland, R.G. 4303 (K) | ERR7621795 | 341 |
| PAFTOL | Convolvulaceae | Ipomoea | nil | Leon, C.J., Lin Y-L, Zhou K -Y 498 (K) | ERR7619226 | 348 |
| SRA | Convolvulaceae | Ipomoea | triloba | NCNSP0323 | ASM357664v1 | 346 |
| PAFTOL | Convolvulaceae | Jacquemontia | paniculata | Barthelat, F. 799 (K) | ERR7621792 | 346 |
| PAFTOL | Convolvulaceae | Lepistemon | owariensis | Mhoro, B. UMBCP 205 (K) | ERR7621801 | 332 |
| PAFTOL | Convolvulaceae | Maripa | scandens | Sothers, C.A.; Pereira, E. da C. 680 (K) | ERR7621765 | 348 |
| PAFTOL | Convolvulaceae | Merremia | pterygocaulos | Pereira, J.K. 2496 (K) | TBC | 345 |
| PAFTOL | Convolvulaceae | Nephrophyllum | abyssinicum | Friis, I.; Bidgood, S.; Hailu, A.; Yitbarew, B. 11498 (K) | ERR7621776 | 344 |
| PAFTOL | Convolvulaceae | Neuropeltis | acuminata | Luke, Q.; Bujo, F. 14809 (K) | ERR7621774 | 349 |
| GAP | Convolvulaceae | Operculina | aequisepala | Westaway, J.O. 3959 (CANB) | ERR7621774 | 349 |
| PAFTOL | Convolvulaceae | Polymeria | ambigua | Trudgen, M.E.; Trudgen, M.; Deluca, S. MET 12302 (K) | ERR7621761 | 345 |
| PAFTOL | Convolvulaceae | Porana | volubilis | Matthew, K.M.; Gastmans, W.F. 72747 (K) | ERR7621777 | 346 |
| PAFTOL | Convolvulaceae | Rapona | tiliifolia | Razakamalala, R.; Rakotovao, C. 5101 (K) | ERR7621789 | 336 |
| PAFTOL | Convolvulaceae | Remirema | bracteata | Staples, G.; Suddee, S.; Simoes, A.R.; Karakey, P. 1338 (K) | ERR7621805 | 316 |
| PAFTOL | Convolvulaceae | Seddera | bagshawei | Friis et al. 9544 (K) | TBC | 348 |
| GAP | Convolvulaceae | Stictocardia | queenslandica | Worboys, S.J. 1586 (K) | ERR7599682 | 342 |
| PAFTOL | Convolvulaceae | Stylisma | pickeringii | Pittman, A.B.; Boyle, K.A. 6229310 (K) | ERR7621810 | 336 |
| PAFTOL | Convolvulaceae | Xenostegia | tridentata | Joel, D.;Tawadong, T. SAN 149920 (K) | ERR7621791 | 351 |
| PAFTOL | Solanaceae | Brunfelsia | brasiliensis | Stehmann, J.R. 6346 (BHCB) | ERR5034091 | 250 |
| PAFTOL | Solanaceae | Coeloneurum | ferrugineum | Santiago-Valentin, E. 93-201 (UPR) | ERR5034081 | 306 |
| PAFTOL | Solanaceae | Duckeodendron | sp. | E. Ribéiro 1189 (K) | ERR7619062 | 280 |
| PAFTOL | Solanaceae | Reyesia | sp. | Wood, J.R.I. SD65 (K) | ERR5084302 | 276 |
| PAFTOL | Solanaceae | Schizanthus | sp. | Knapp, S. et al 10516 (BM) | ERR7619053 | 303 |
| PAFTOL | Montiniaceae | Grevea | eggelingii | Robertson 3643 (K) | ERR4180037/  ERR5010320 | 308 |

*PAFToL = Plant and Fungal Tree of Life; GAP = Genomics for Australian Plants; SRA = Sequence Read Archive.
